# Metagenomic discovery of novel CRISPR-Cas13 systems

**DOI:** 10.1038/s41421-022-00464-5

**Published:** 2022-10-11

**Authors:** Yanping Hu, Yangcan Chen, Jing Xu, Xinge Wang, Shengqiu Luo, Bangwei Mao, Qi Zhou, Wei Li

**Affiliations:** 1 Savaid Medical School, University of Chinese Academy of Sciences, Beijing, China; 2 State Key Laboratory of Stem Cell and Reproductive Biology, Institute of Zoology, Chinese Academy of Sciences, Beijing, China; 3 Institute for Stem Cell and Regenerative Medicine, Chinese Academy of Sciences, Beijing, China; 4 Bejing Institute for Stem Cell and Regenerative Medicine, Beijing, China

**Keywords:** Bioinformatics, Cell biology

Dear Editor,

CRISPR-Cas systems are crucial adaptive immune components of microbial resistance against the invasion of mobile genetic elements (MGEs) and serve as the core of current cutting-edge genome engineering technologies^1^. Unlike the widely applied Cas9 or Cas12 DNA editing tools in present use, Cas13 is an RNA-guided programable RNA-targeting single effector system that enables gene manipulation at the transcriptional level^2^. At present, only four subtypes of Cas13 have been identified^1,3,4^. An expanded catalog of CRISPR-Cas13 systems can provide phylogenetic insights and may offer opportunities for the development of novel RNA-editing tools. By mining bulk metagenomic data (> 10 TB) from various environments, we identified hundreds of orthologs of known and novel Cas13 systems in this study, the latter of which could be classified into five novel subtypes based on protein sequence similarity. Notably, the novel Cas13 systems discovered in this study can be developed into efficient RNA editors and expand the RNA-editing toolbox.

In this study, we developed a computational pipeline for the de novo identification of novel Cas13 proteins (Fig. 1a). Initially, putative CRISPR arrays were identified from sequenced data. Then, 20 kb regions of DNA flanking the CRISPR arrays were extracted for predicting the open reading frames (ORFs). Proteins with more than 400 residues were selected for further analyses. A Cas13 library consisting of profile hidden Markov models (HMMs) of all known Cas13a, Cas13b, Cas13c, and Cas13d protein sequences in the NCBI database was subsequently constructed^5^. We proposed that the use of this library, which includes the features of all known Cas13 proteins, could maximize the possibility of discovering potential novel Cas13 proteins from uncharacterized protein sequences. The identified proteins that were devoid of two higher eukaryotes and prokaryotes nucleotide-binding (HEPN) domains were assumed to be incomplete and less likely to be active^6^ and were therefore not selected for further analyses. Novel Cas13 proteins were further defined based on the results of phylogenetic analyses^1,4^.

**Fig. 1:**
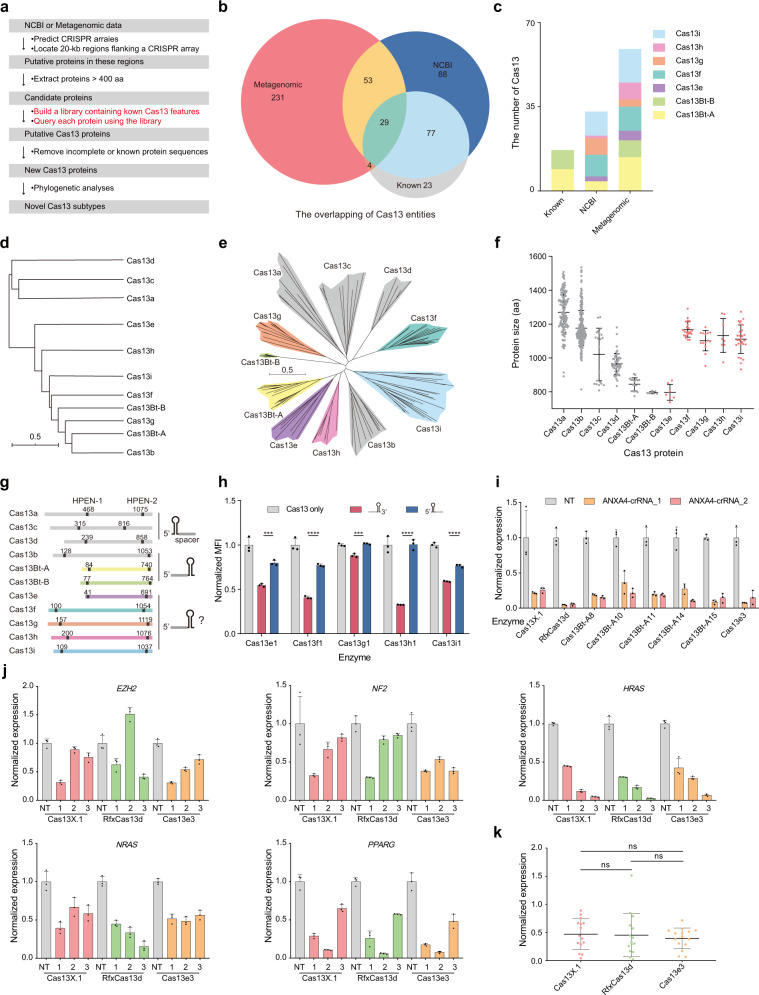
Identification of CRISPR-Cas13 system and RNA-degrading capacity of the novel Cas13 protein. **a** Description of the bioinformatics pipeline used for identifying novel Cas13 families. **b** Overlapping of novel Cas13 proteins with proteins of previously discovered Cas13 subtypes, which are known or unannotated in the NCBI and metagenomic databases. **c** Distribution of the number of novel Cas13 subtypes. **d** Dendrogram constructed using the unweighted pair group method with arithmetic mean (UPGMA) algorithm. From top to bottom, *n* = 502, 51, 411, 7, 8, 27, 22, 8, 14, 18, and 925. **e** Maximum likelihood tree of the Cas13 families. **f** Distribution of the size of the proteins in the different Cas13 subtypes. From left to right, *n* = 151, 281, 21, 52, 18, 8, 7, 22, 14, 8, and 27. **g** Schematic diagram depicting the size of the Cas13 proteins and the position of the HEPN motif. **h** Comparison of the gene knockdown efficiency using crRNAs with 3′- or 5′-DR sequences (means ± s.d.; *n* = 3 biological replicates; Student’s *t*-test, ns not significant, ***P < 0.001, ****P < 0.0001). Normalized MFI, mean fluorescence intensity (MFI) relative to the non-targeting condition. **i** Measurement of *ANXA4* mRNA knockout mediated by the novel Cas13 systems in HEK293T cells using quantitative reverse transcription PCR (RT-qPCR) (means ± s.d.; *n* = 3 biological replicates). Normalized expression, relative expression was normalized to the non-targeting condition. NT non-targeting crRNA. **j** Evaluation of the cleavage activity of Cas13 proteins by targeting the mRNAs of endogenous genes using RT-qPCR in HEK293T cells (means ± s.d.; *n* = 3 biological replicates). Normalized expression, relative expression was normalized to the non-targeting condition. NT non-targeting crRNA. **k** Comparison of the endogenous gene knockdown activity of Cas13X.1, RfxCas13d, and Cas13e3 (means ± s.d.; *n* = 15 for each protein; paired *t*-test, ns not significant). Normalized expression, relative expression was normalized to the non-targeting condition.

In order to test the accuracy of the bioinformatics pipeline developed herein, the pipeline was initially used to search for previously discovered Cas13 proteins in the NCBI’s prokaryotic database. As expected, previously discovered Cas13 systems were successfully identified using the pipeline (Supplementary Fig. S1a). We next searched for novel Cas13 proteins from metagenomic databases containing data from host-associated, aquatic, and soil environments (Supplementary Tables S1–S6). Compared to the commonly accessible data in the NCBI database, metagenomic data enables the identification of a greater number of novel Cas13 proteins (Fig. 1b, c and Supplementary Fig. S1b–d), demonstrating the importance and necessity of metagenomic mining. The protein sequences were next subjected to phylogenetic analyses, and the results demonstrated that the novel Cas13 proteins clustered into seven new branches distinct from those of the known subtypes (Fig. 1d)^1^. Of these seven clades, two clades, namely, Cas13Bt-A and Cas13Bt-B, with an approximate average sequence length of 800 amino acids (aa), could be clustered into the same clades as the previously discovered Cas13X, Cas13Y, and Cas13bt proteins (Fig. 1e)^3,4^. The remaining five clades were designated as Cas13e to Cas13i, with protein lengths ranging from 740 to 1300 aa (Fig. 1d–f). Specifically, the average lengths of Cas13e and Cas13f-i proteins were ~800 and 1100 aa, respectively (Fig. 1f).

Analysis of the CRISPR locus revealed that with the exception of Cas13g, all the novel Cas13 subtypes discovered herein lacked conserved adaptation genes, including *Cas1* and *Cas2* (Supplementary Fig. S1e). We next analyzed the features in the CRISPR array. Like other CRISPR-Cas13 systems^7,8^, the average lengths of the spacers and direct repeats (DRs) of the novel Cas13 systems are 30 and 36 nt, respectively (Supplementary Fig. S2a, b). Multiple sequence alignment of the DRs revealed that they are highly conserved and have similar predicted secondary RNA structures, which were similar to the characteristics of the DR sequences of previously identified Cas13a, Cas13b, and Cas13d systems (Supplementary Fig. S2a, b)^6,9,10^. Using the spacer sequences as a query, the potential targets of natural crRNA from the CRISPR locus were investigated by searching the IMG/VR, Genbank-Phage, and Ref-Plasmid databases. Positive hits indicated that these CRISPR-Cas13 systems could be active in hosts and defend against foreign MGEs.

We next analyzed the features of the sequences of the novel Cas13 proteins. Multiple sequence alignment of these novel proteins of each subtype revealed that their HEPN domains were highly conserved, and the RNXXXH motif was most conserved, accounting for ~74% of all RXXXXH motifs (Supplementary Fig. S3a, b). We observed that only Cas13a, Cas13c, and Cas13d possessed an elongated N-terminal domain (NTD) (Fig. 1g)^6,9–11^. The existing structure of Cas13 revealed that Cas13a and Cas13d contain an NTD at the N-terminus, which is the least conserved region in Cas13 proteins and forms a binding channel for the DR region^9,11^; however, this domain is absent in Cas13b^10^. Interestingly, while Cas13a, Cas13c, and Cas13d systems use mature crRNAs with a 5′-DR sequence for effective RNA interference, we noticed that the mature crRNAs of Cas13b, Cas13Bt-A, and Cas13Bt-B systems contained a 3′-DR sequence^3,4,7,8,12^. Based on this consistency, we speculated whether the existence of the NTD domain of the novel CRISPR-Cas13 systems could be used for predicting the optimal configuration of the crRNA for effective RNA inference. Using this hypothesis, we deduced that the novel Cas13e, Cas13f, Cas13g, Cas13h, and Cas13i systems, with a 3′-DR sequence in the crRNA, would be more efficient in cleaving RNA (Supplementary Fig. S4a). We employed a mammalian cell-based mCherry disruption system for evaluating the RNA cleavage activity of the novel Cas13 proteins (Supplementary Fig. S4b). Consistent with our speculation, we observed that the use of crRNAs with 3′-DR sequences, and not 5′-DR sequences, enabled the effective disruption of the mCherry mRNA by Cas13e1, Cas13f1, Cas13g1, Cas13h1, and Cas13i1 (Fig. 1h and Supplementary Fig. S4a). Collectively, the lack of the NTD domain in Cas13 can reflect on the structural differences of its cognate mature crRNA, and this rule can be used when employing novel Cas13 proteins with little known structural information for RNA editing in mammalian cells.

The clinical application of Cas13-based RNA-editing systems continues to be challenging at present, partly due to the fact that the large size of RNA editors exceeds the packaging capacity of adeno-associated virus (AAV) vectors. The hypercompact Cas13 proteins discovered in this study can aid in overcoming this limitation. In order to identify novel small Cas13 systems with high activity, the mCherry reporter system was used for initial screening (Supplementary Fig. S4b, c). The mCherry signals were substantially reduced using the novel Cas13 proteins (Supplementary Fig. S4d). To verify the endogenous gene knockdown activity of the novel Cas13 proteins, two sites in the *ANXA4* gene were selected for testing in HEK293T cells (Fig. 1i). Of note, Cas13e3 achieved the highest knockdown activity among all the Cas13 systems screened herein, with the efficiency at the two sites being 92% and 84% (Fig. 1i). We, therefore, selected Cas13e3 for further characterization owing to its high efficiency and the ultrasmall protein size (767 aa).

In order to investigate the optimal length of the spacer for Cas13e3 editing, we tested two different crRNAs for targeting the mCherry mRNA, with spacers of lengths ranging from 5 to 50 nt (Supplementary Fig. S5a). Cas13e3 achieved the highest average knockdown efficiency at the two selected sites when the 27-nt spacer was used (Supplementary Fig. S5a). The 27-nt spacer was therefore used for subsequent experiments. We next sought to determine the protospacer flanking sequence (PFS) requirement of Cas13e3. The results of screening revealed no PFS preference for Cas13e3 (Supplementary Fig. S5b). Besides, we observed that fusion with the nuclear localization signal (NLS) could increase the knockdown efficiency of Cas13e3 (Supplementary Fig. S5c). In order to investigate the knockdown efficiency of Cas13e3 on a larger scale, a total of 15 crRNAs targeting five genes were tested (Fig. 1j). We also tested the activities of Cas13X.1 and RfxCas13d at the same sites for comparison. Notably, we observed that Cas13e3 exhibited robust knockdown activity that was comparable to that of Cas13X.1 and RfxCas13d (Fig. 1j, k). We further investigated the specificity of Cas13e3 on a genome-wide scale. A total of 102, 323, and 133 differentially expressed genes were detected using RNA-Seq for the Cas13X.1, RfxCas13d, and Cas13e3 systems, respectively (Supplementary Fig. S6a), indicating that the specificity of Cas13e3 was comparable to that of Cas13X.1, while its off-target effect was lower than that of RfxCas13d (Supplementary Fig. S6a). In order to compare the collateral RNA cleavage activities of Cas13X.1, RfxCas13d, and Cas13e3, the fluorescence intensity of enhanced green fluorescent protein (EGFP) was measured when targeting the mCherry mRNA^13^. Notably, collateral RNA cleavage activities were not detected for Cas13X.1 and Cas13e3, while RfxCas13d showed dramatic trans-cleavage activity for EGFP transcripts (Supplementary Fig. S6b). In conclusion, these results demonstrated that Cas13e3 is a hypercompact RNA editor with high interference efficiency and low collateral activity.

In this study, we developed a computational pipeline to sensitively discover novel CRISPR systems by constructing a library containing comprehensive Cas13 features. By mining metagenomic data, we identified five novel Cas13 clades. We verified that several of the novel Cas13 families have RNA-degrading capacity in mammalian cells. Importantly, the novel Cas13e3 protein discovered in this study is an ultracompact Cas13 protein, and can be developed into an efficient transcriptome editor in mammalian cells. The novel systems identified in this study substantially increase the diversity of CRISPR-Cas13 systems and largely expand the programmable RNA-editing toolbox.

## Supplementary information

Supplementary Methods and Figures

Supplementary Tables

## Data Availability

The sequencing data have been deposited in Genome Sequence Archive for human (GSA-Human) of Beijing Institute of Genomics, Chinese Academy of Sciences (https://ngdc.cncb.ac.cn/gsa-human/). The accession number is HRA002695.
