# Supplementary material for: Metagenomic discovery of novel CRISPR-Cas13 systems
Source: Cell Discov. 2022 Oct 11;8:107. doi: 10.1038/s41421-022-00464-5 (PMC9554183; doi:10.1038/s41421-022-00464-5)
Supplement: Supplementary file 1 — Supplementary Methods and Figures [file 41421_2022_464_MOESM1_ESM.pdf]

Supplementary Information for

Metagenomic discovery of Novel CRISPR-Cas13 systems

Yanping Hu, Yangcan Chen, Jing Xu, Xinge Wang, Shengqiu Luo, Bang-wei Mao, Qi Zhou*, Wei Li*

Correspondence to: Wei Li (liwei@ioz.ac.cn), Qi Zhou (zhouqi@ioz.ac.cn)

This PDF file includes:

Materials and Methods

Supplementary Figs. S1 to 6

Captions for Supplementary Tables

Other Supplementary Material for this manuscript includes the following:

Supplementary Tables (.xls)

Materials and Methods

Identification of novel Cas13 systems

Over 10 TB of assembled metagenomic sequences were retrieved from the Joint Genome Institute (JGI)[1,2], NCBI[3], and European Nucleotide Archive (ENA)[4] databases. The CRISPR arrays were identified using the MinCED software[5] with default parameters. Two 20 kb DNA regions flanking the CRISPR array were extracted for protein prediction. ORFs larger than 400 aa were annotated using Meta-GeneMark 1[6]. The protein sequences of all known subtypes from Cas13a to Cas13d were retrieved from NCBI, and used to generate HMMs using the HH-suite[7] for constructing the Cas13 library. The library was then used to align the newly discovered proteins for identifying the potential novel Cas13 sequences. Incomplete or known proteins were removed to enable detection of the intact and novel Cas13 sequences.

Phylogenetic analyses

For phylogenetic analyses and classification, the candidate sequences were clustered using MMseqs2[8] using the following criteria: sequence identity of 0.5 and minimum coverage of 0.7. The proteins within each cluster were clustered using MMseqs2[8] with the following parameters for reducing redundancy: sequence identity of 0.9 and minimum coverage of 0.8. Each non-redundant cluster was aligned using MAFFT[9], with default parameters. The aligned non-redundant clusters were converted into profile HMMs using HHmake[7], and the profile HMMs were next aligned using the HMM-HMM alignment method in HHalign[7]. The resulting alignment score between clusters was s_{ij}, where i and j represent clusters i and j, respectively. The average score was first calculated using the function $S_{ij} = (s_{ij}+s_{ji})/2$. The pseudo-distance was next calculated using the function $d_{ij} = (\log(\min(S_{ii},S_{jj}))-\log(S_{ij}))/2$. These distances were used for constructing a

dendrogram with the UPGMA algorithm for classification.

The protein sequences were aligned using MUSCLE[10]. The phylogenetic tree was constructed with FastTree[11] and visualized using the online iTOL tool[12]. Owing to the inaccuracy of the metagenomic data during sequencing and assembly, the CD-HIT software was used to remove redundant sequences with a sequence identity threshold of 80% prior to determining the number of proteins[13,14]. The consensus secondary structures and RNA secondary structures were predicted using RNAalifold and RNAfold webservers, respectively[15]. The RNA sequence logos were generated with WebLogo[16]. CRISPSRTarget[17] was used to search for the potential targets of natural crRNA and the positive hits are enlisted in Supplementary Table S4.

Vector construction

The human codon-optimized sequences of Cas13 enlisted in Supplementary Table S1 were synthesized *de novo* by GenScript. The DNA sequences encoding the Cas13 proteins were cloned into the pCAG-2A-EGFP backbone digested with XmaI and NheI[18], using the Gibson assembly method. The sequence, which consisted of the U6 promoter and crRNA, was inserted into the pUC19 backbone digested with EcoRI and HindIII. The sequences of the crRNA scaffolds and spacers used in this study are enlisted in Supplementary Tables S2 and S3. The mCherry sequence was cloned downstream of the EF-1α promoter for generating the EF-1α-mCherry plasmid for the mCherry reporter system.

Mammalian cell culture and transfection

HEK293T cells were cultured in Dulbecco’s modified Eagle’s medium (DMEM, Gibco) supplemented with 10% fetal bovine serum (FBS, Gibco) and 1% penicillin-streptomycin (Gibco). For transfection, the cells were plated in a 24-well dish (Corning) until they reached a confluence

of 90%. Lipofectamine 3000 Reagent (Invitrogen) was used for all the transfections.

mCherry disruption system used in mammalian cells

The mCherry disruption system was used to evaluate the mRNA cleavage activity of the Cas13 proteins. To this end, Cas13 and EGFP proteins were co-expressed from the CAG promoter (designated as CAG-Cas13), while its cognate mature crRNA targeting the mCherry mRNA was expressed by the U6 promoter (designated as U6-crRNA). The mCherry was expressed by the EF-1α promoter cloned in a separate plasmid (designated as EF-1α-mCherry). The three vectors were transfected into human HEK293T cells using Lipofectamine 3000 Reagent (Invitrogen) according to the manufacturer's instructions. The mean fluorescence intensity (MFI) of EGFP and mCherry double-positive cells was calculated 48 hours post-transfection by flow cytometry.

In order to verify the configuration of the crRNA generating efficient RNA knockdown efficiency, the U6-crRNA plasmid expressing crRNA with 5′-DR or 3′-DR sequences was constructed. The MFI was normalized to that of the negative control, comprising HEK293T cells transfected with CAG-Cas13 and EF-1α-mCherry plasmids.

In order to analyze the PFS preference of Cas13e3, targets flanking 16 kinds of PFS combinations were cloned upstream of the mCherry gene in the EF-1α-mCherry plasmid. The MFI was normalized to that of the negative control, comprising HEK293T cells transfected with the CAG-Cas13 plasmid, U6-crRNA plasmid, and EF-1α-mCherry plasmid without PFS targets.

Mammalian mRNA knockdown assays

For the mammalian mRNA knockdown experiments, HEK293T cells were transfected with 600 ng of a plasmid expressing Cas protein and 300 ng of a plasmid encoding crRNA. HEK293T cells were lysed with TRIzol® reagent (Life Technologies) 48 hours post-transfection, and the total

RNAs were extracted with a PureLink™ RNA Mini Kit (Thermo Fisher). Finally, quantitative reverse transcription PCR (RT-qPCR) was performed using a HiScript® II One Step qRT-PCR SYBR Green Kit (Vazyme). The sequences of the primers used for RT-qPCR are enlisted in Supplementary Table S5. The results of RT-qPCR were analyzed using the $2^{-\Delta\Delta CT}$ method. Relative expression was normalized to that of a negative control comprising HEK293T cells transfected with a plasmid expressing Cas13 protein and a plasmid encoding non-targeting (NT) crRNA.

RNA-seq analysis

The total RNA extracted from each sample was subjected to RNA-seq analysis using an Illumina NextSeq system. The sequencing reads were mapped onto the human genome (GRCh38) using the Hisat2 (v2.2.1) software[19], and the reads were next counted using the htseq-count software[20]. The read counting results were used for calculating the fragments per kilobase million (FPKM). The differentially expressed genes were identified using the DEseq2 package[21]. Genes with a fold change greater than 2 and false discovery rate (FDR) less than 0.05 were regarded as differentially expressed genes.

Statistical analyses

The statistical analyses enlisted in Supplementary Table S6 were performed using GraphPad Prism software version 8.0.

References

1 Chen, I. A. et al. The IMG/M data management and analysis system v.6.0: new tools and advanced capabilities. Nucleic Acids Res 49, D751-D763, doi:10.1093/nar/gkaa939 (2021).

2 Nordberg, H. et al. The genome portal of the Department of Energy Joint Genome Institute: 2014 updates. Nucleic Acids Res 42, D26-31, doi:10.1093/nar/gkt1069 (2014).

3 Benson, D. A. et al. GenBank. Nucleic Acids Res 41, D36-42, doi:10.1093/nar/gks1195 (2013).

4 Stewart, R. D. et al. Compendium of 4,941 rumen metagenome-assembled genomes for rumen microbiome biology and enzyme discovery. Nat Biotechnol 37, 953-961, doi:10.1038/s41587-019-0202-3 (2019).

5 Bland, C. et al. CRISPR recognition tool (CRT): a tool for automatic detection of clustered regularly interspaced palindromic repeats. BMC Bioinformatics 8, 209, doi:10.1186/1471-2105-8-209 (2007).

6 Besemer, J. & Borodovsky, M. Heuristic approach to deriving models for gene finding. Nucleic Acids Res 27, 3911-3920, doi:10.1093/nar/27.19.3911 (1999).

7 Steinegger, M. et al. HH-suite3 for fast remote homology detection and deep protein annotation. BMC Bioinformatics 20, 473, doi:10.1186/s12859-019-3019-7 (2019).

8 Steinegger, M. & Söding, J. MMseqs2 enables sensitive protein sequence searching for the analysis of massive data sets. Nature Biotechnology 35, 1026-1028, doi:10.1038/nbt.3988 (2017).

9 Katoh, K. & Standley, D. M. MAFFT multiple sequence alignment software version 7: improvements in performance and usability. Mol Biol Evol 30, 772-780, doi:10.1093/molbev/mst010 (2013).

10 Edgar, R. C. MUSCLE: multiple sequence alignment with high accuracy and high throughput. Nucleic Acids Res 32, 1792-1797, doi:10.1093/nar/gkh340 (2004).

11 Price, M. N., Dehal, P. S. & Arkin, A. P. FastTree 2--approximately maximum-likelihood trees for large alignments. PLoS One 5, e9490, doi:10.1371/journal.pone.0009490 (2010).

12 Letunic, I. & Bork, P. Interactive Tree Of Life (iTOL) v5: an online tool for phylogenetic tree display and annotation. Nucleic Acids Res 49, W293-W296, doi:10.1093/nar/gkab301 (2021).

13 Li, W. & Godzik, A. Cd-hit: a fast program for clustering and comparing large sets of protein or nucleotide sequences. Bioinformatics 22, 1658-1659, doi:10.1093/bioinformatics/btl158 (2006).

14 Fu, L., Niu, B., Zhu, Z., Wu, S. & Li, W. CD-HIT: accelerated for clustering the next-generation sequencing data. Bioinformatics 28, 3150-3152, doi:10.1093/bioinformatics/bts565 (2012).

15 Gruber, A. R., Bernhart, S. H. & Lorenz, R. The ViennaRNA web services. Methods Mol Biol 1269, 307-326, doi:10.1007/978-1-4939-2291-8_19 (2015).

16 Crooks, G. E., Hon, G., Chandonia, J. M. & Brenner, S. E. WebLogo: a sequence logo generator. Genome Res 14, 1188-1190, doi:10.1101/gr.849004 (2004).

17 Biswas, A., Gagnon, J. N., Brouns, S. J., Fineran, P. C. & Brown, C. M. CRISPRTarget: bioinformatic prediction and analysis of crRNA targets. RNA Biol 10, 817-827,

doi:10.4161/rna.24046 (2013).
18 Teng, F. et al. Repurposing CRISPR-Cas12b for mammalian genome engineering. Cell Discovery 4, 63, doi:10.1038/s41421-018-0069-3 (2018).
19 Kim, D., Langmead, B. & Salzberg, S. L. HISAT: a fast spliced aligner with low memory requirements. Nat Methods 12, 357-360, doi:10.1038/nmeth.3317 (2015).
20 Anders, S., Pyl, P. T. & Huber, W. HTSeq--a Python framework to work with high-throughput sequencing data. Bioinformatics 31, 166-169, doi:10.1093/bioinformatics/btu638 (2015).
21 Love, M. I., Huber, W. & Anders, S. Moderated estimation of fold change and dispersion for RNA-seq data with DESeq2. Genome Biol 15, 550, doi:10.1186/s13059-014-0550-8 (2014).

Supplementary Fig. 1

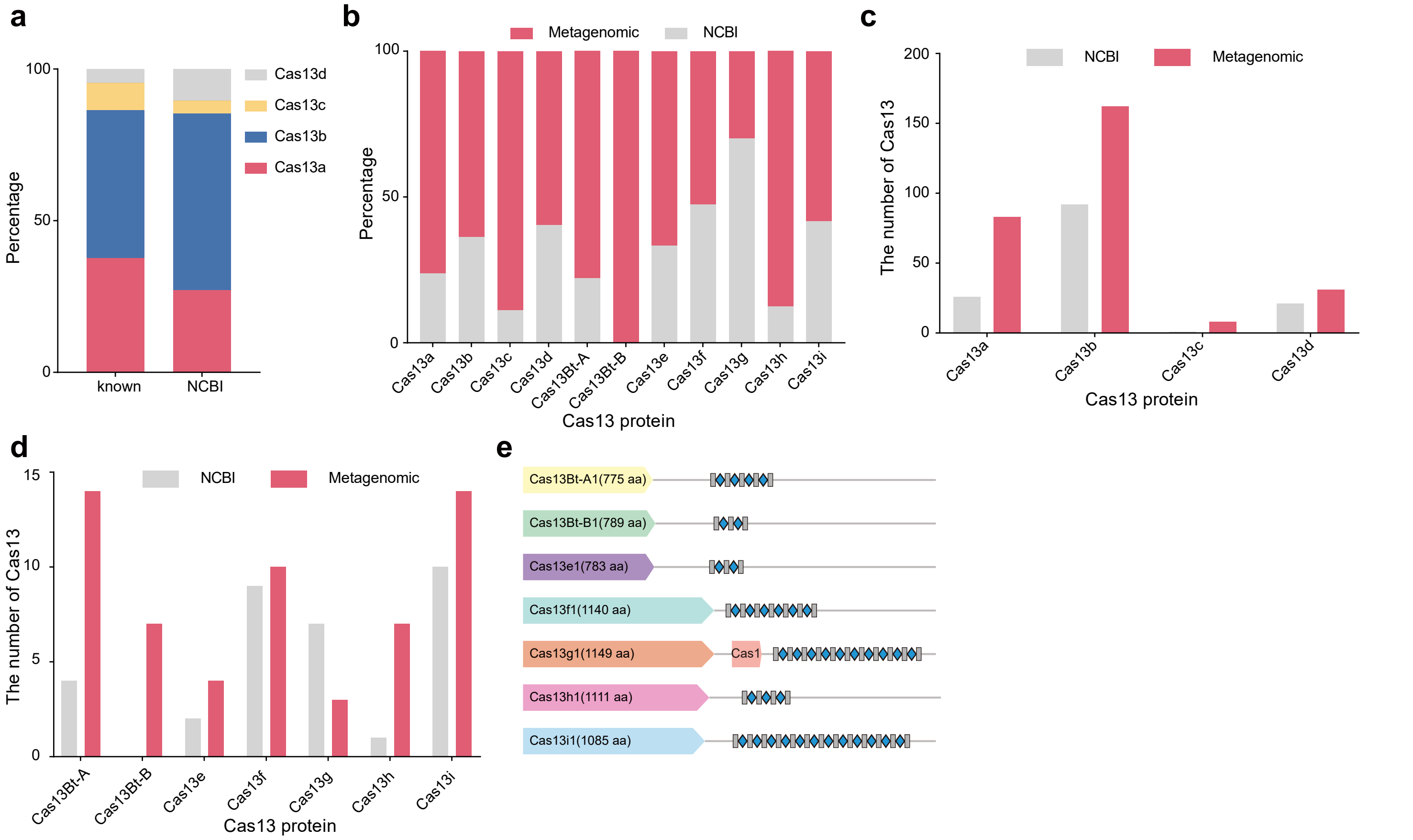

Supplementary Fig. 1 Analysis of all the novel Cas13 proteins identified in this study.
a. Distribution of echo type of previously discovered Cas13 subtypes, known and unannotated in the NCBI database.
b. Distribution of novel proteins belonging to different Cas13 subtypes in the NCBI and metagenomic databases.
c. Number of novel orthologs of known subtypes in the NCBI and metagenomic databases.
d. Number of novel Cas13 systems in the NCBI and metagenomic databases.
e. Loci organization of novel Cas13 systems.

Supplementary Fig. 2

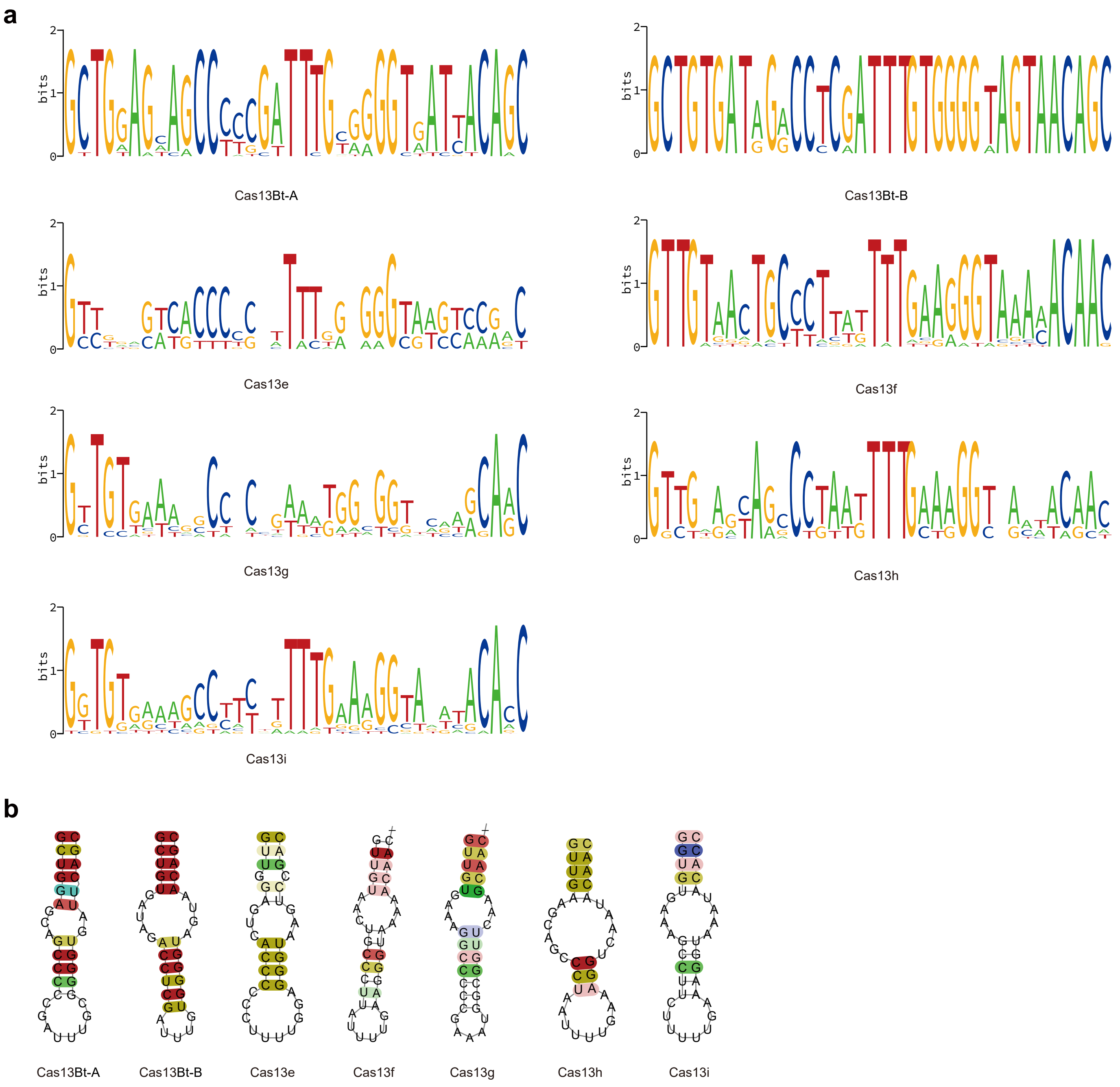

Supplementary Fig. 2 Analyses of DR sequences.

a. Sequence logos of the DR sequences of the novel Cas13 proteins in each subtype.

b. Consensus secondary structure of the DRs of the novel Cas13 proteins in each subtype was predicted using the RNAalifold webserver.

Supplementary Fig. 3

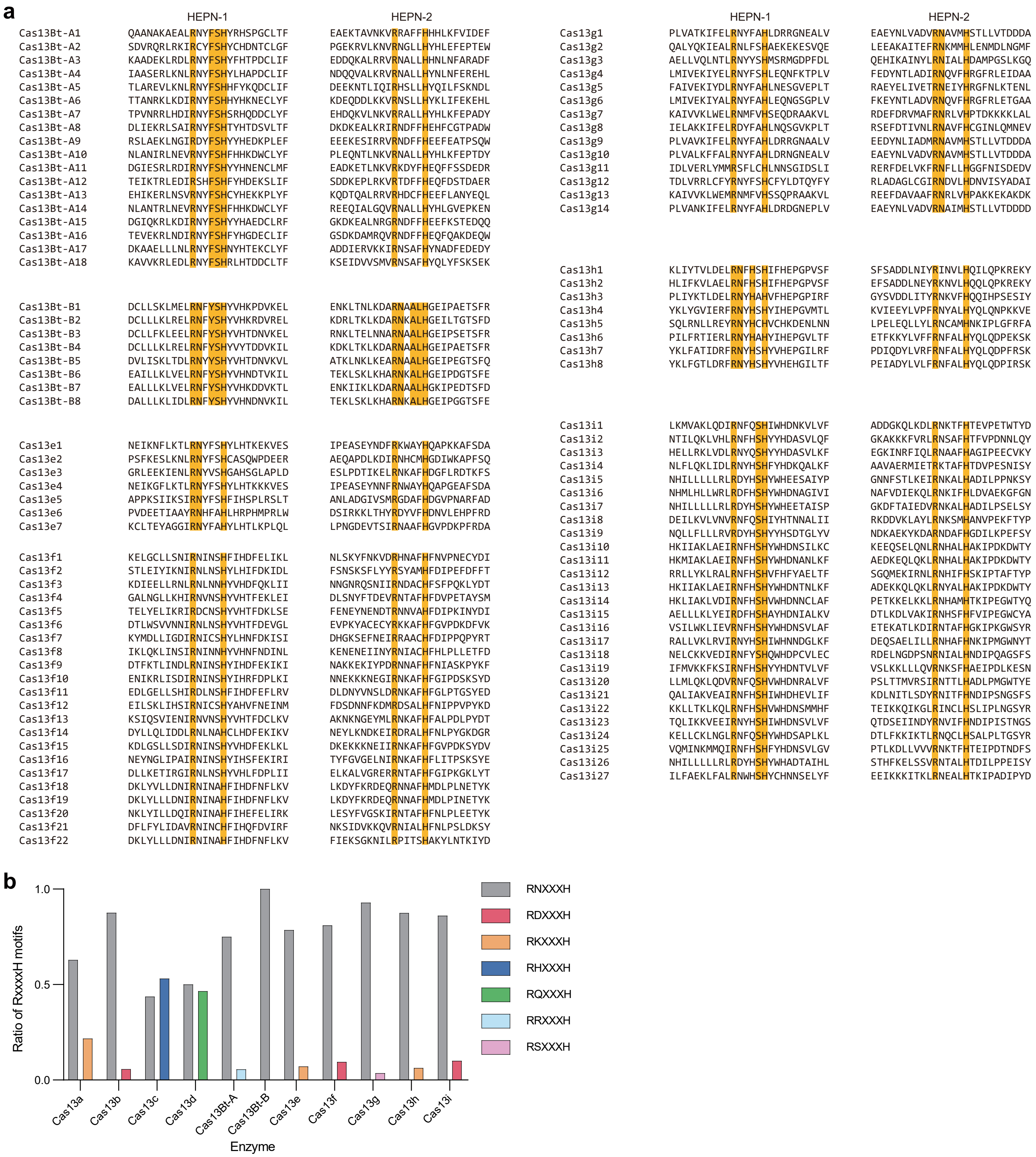

Supplementary Fig. 3 Analyses of HEPN motifs.

a. Alignment of the HEPN domains of the novel Cas13 proteins of each subtype. The conserved residues have been highlighted in orange.

b. Distribution of different HEPN motifs among different Cas13 subtypes.

Supplementary Fig. 4

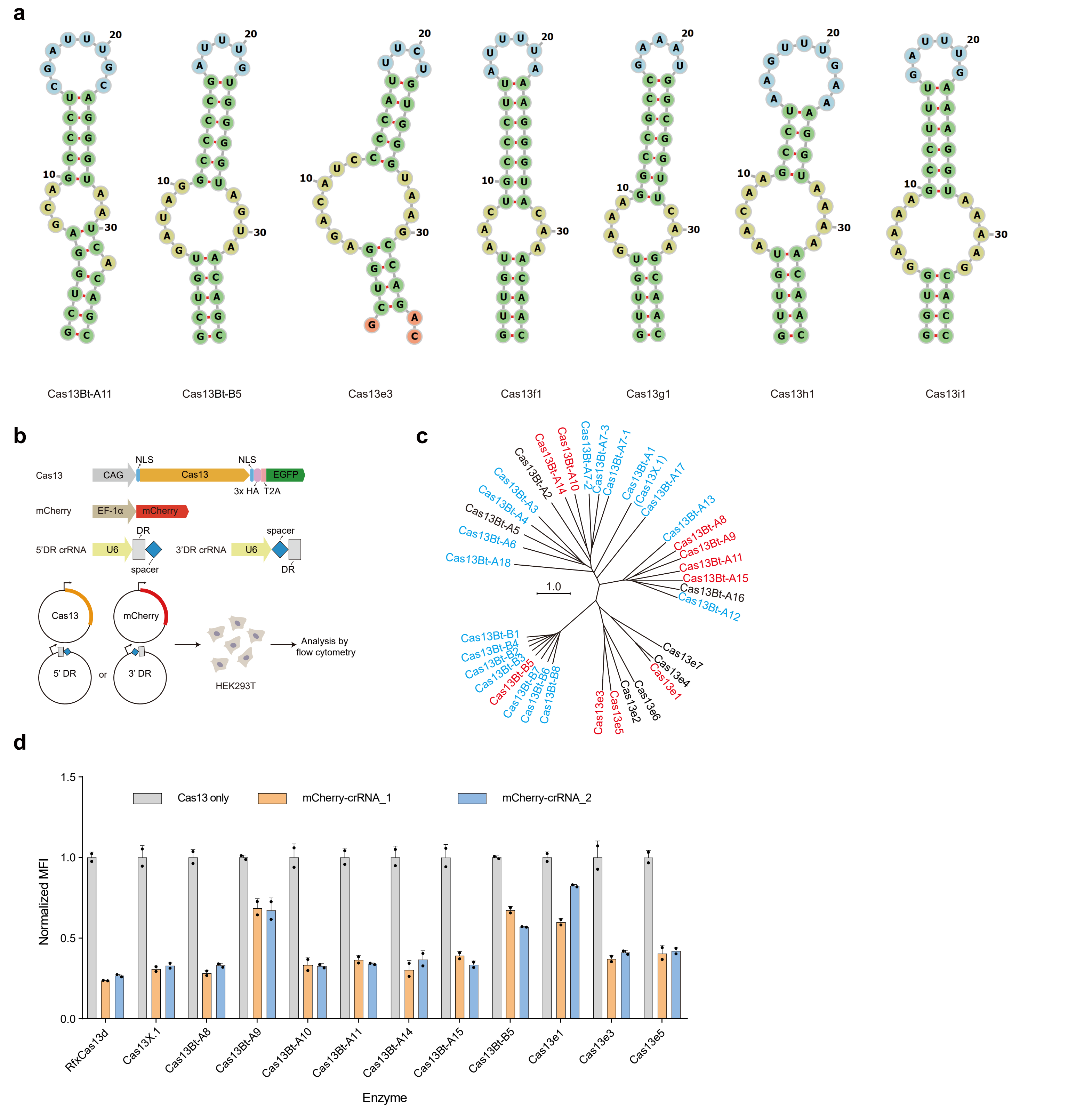

Supplementary Fig. 4 Identification of RNA-degrading capacity of compact Cas13 proteins.
a. Predicted RNA secondary structure of the DR sequence of a representative Cas13 system. The RNA secondary structures were predicted using the RNAfold webserver.
b. Experimental procedure of the mammalian cell-based mCherry reporter system. CAG, CAG promoter. NLS, nuclear localization signal. 3x HA, 3 x Hemagglutinin (HA) Tag. T2A, T2A self-cleaving peptide. EGFP, enhanced green fluorescent protein. EF-1α, EF-1α promoter. mCherry, mCherry fluorescent protein. U6, U6 promoter.
c. Maximum likelihood tree of the small Cas13 proteins. The previously discovered Cas13 proteins have been highlighted in blue. The experimentally studied novel Cas13 proteins have been marked in red.
d. Screening of Cas13 proteins with higher knockdown efficiency in the mammalian cell-based mCherry reporter system (mean ± s.d.; n = 2 biological replicates). Normalized MFI, mean fluorescence intensity (MFI) relative to the nontargeting condition.

Supplementary Fig. 5

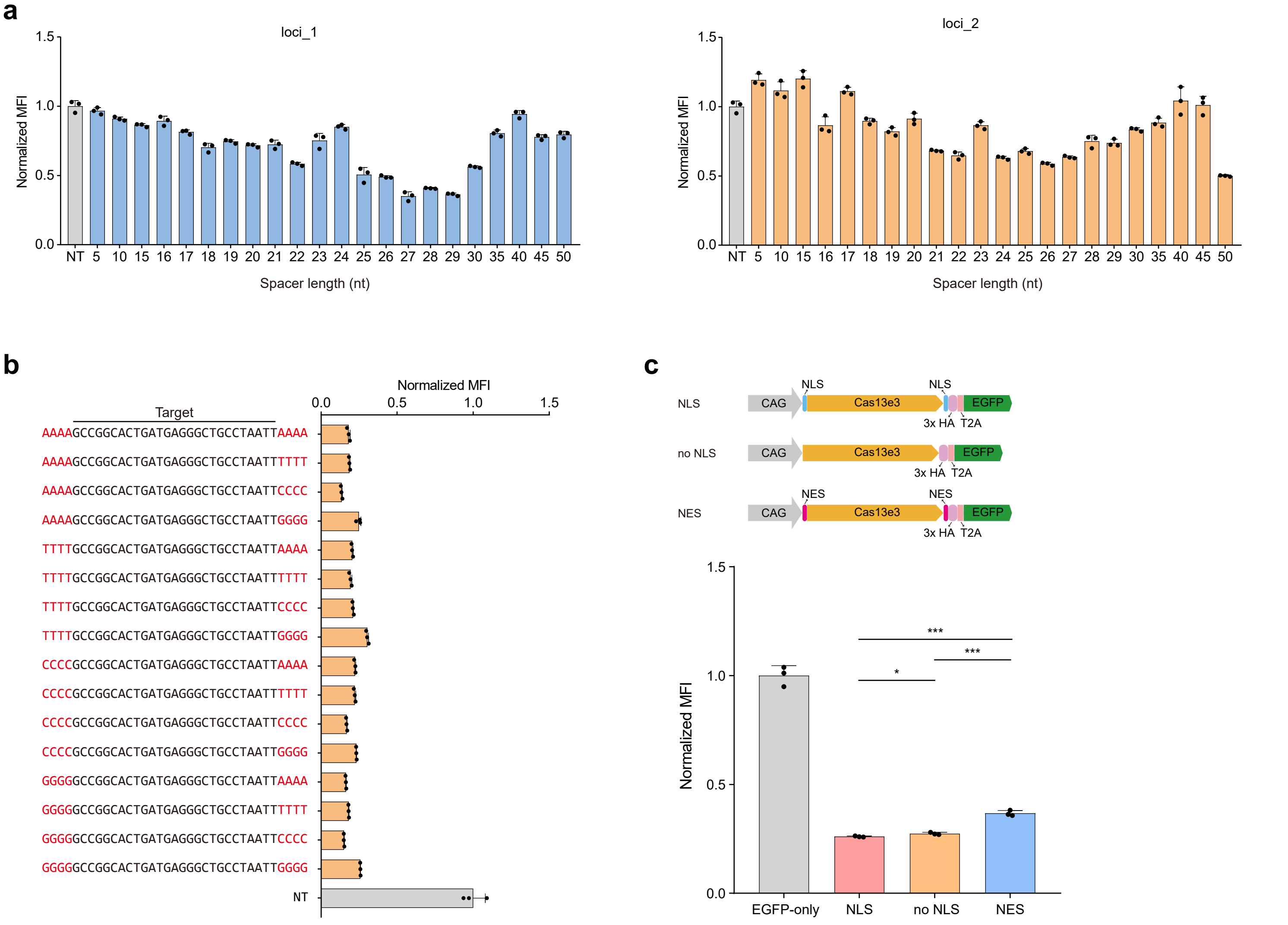

Supplementary Fig. 5 Effects of spacer length, PFS, and NLS on Cas13e3 activity.

a. Variations in mCherry fluorescence intensity using different crRNAs with spacers of lengths ranging between 5 and 50 nt (mean ± s.d.; n = 3 biological replicates). Normalized MFI, MFI relative to the nontargeting condition. NT, non-targeting crRNA. nt, nucleotide.

b. PFS preference analysis for different PFS-flanking crRNAs (mean ± s.d.; n = 3 biological replicates). Normalized MFI, MFI relative to the nontargeting condition. NT, non-targeting crRNA.

c. The upper panel shows a schematic representation of the different plasmid constructs encoding the engineered Cas13e3 protein. The lower panel shows a comparison of the different localization signals for mCherry mRNA knockdown (mean ± s.d.; n = 3 biological replicates). Normalized MFI, MFI was normalized to the nontargeting condition. CAG, CAG promoter. NLS, nuclear localization signal. no NLS, without NLS. 3xHA, 3 x Hemagglutinin (HA) Tag. T2A, T2A self-cleaving peptide. EGFP, enhanced green fluorescent protein. NES, nuclear export sequence.

Supplementary Fig. 6

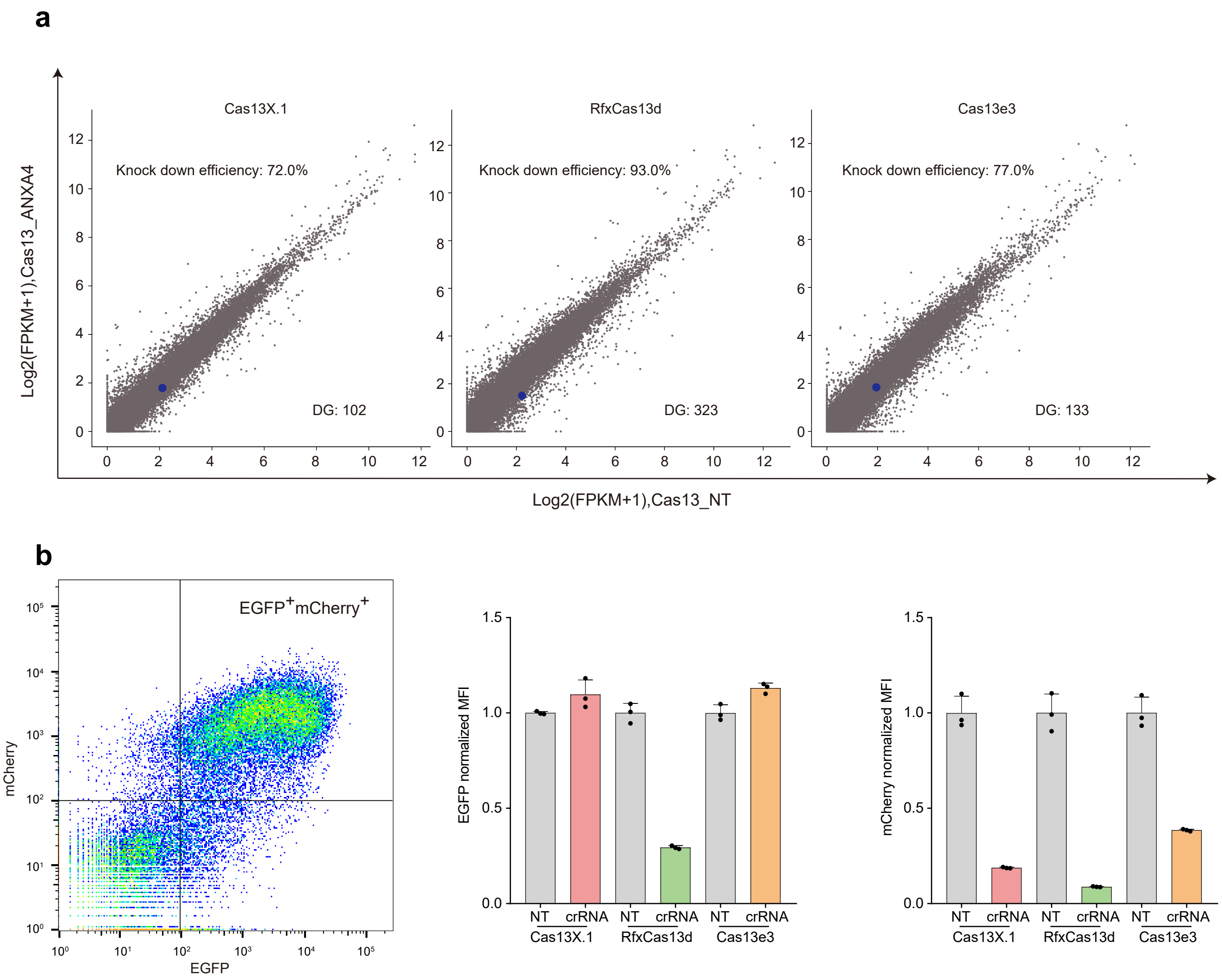

Supplementary Fig. 6 Comparison of the specificity and collateral RNA cleavage activity of Cas13X.1, RfxCas13d, and Cas13e3.

a. RNA-seq analysis showing the changes in gene expression following ANXA4 mRNA knockdown in HEK293T cells by Cas13X.1, RfxCas13d, and Cas13e3 (mean ± s.d.; n = 2 biological replicates). DG, downregulated genes. NT, non-targeting crRNA. FPKM, fragments per kilobase million.

b. Comparison of the collateral RNA cleavage activity of Cas13X.1, RfxCas13d, and Cas13e3 by targeting the mCherry mRNA and evaluation of EGFP fluorescence intensity (mean ± s.d.; n = 3 biological replicates). Normalized MFI, MFI relative to the nontargeting condition. NT, non-targeting crRNA. EGFP, enhanced green fluorescent protein.

Captions for Supplementary Tables.

Table S1. The sequences and sources of Cas13 proteins.

Table S2. The sequences of DRs.

Table S3. The sequences of mRNA spacers.

Table S4. The CRISPRTarget results of natural spacers.

Table S5. The sequences of RT-qPCR primers.

Table S6. Statistics.
